# Queen Quality and the Impact of Honey Bee Diseases on Queen Health: Potential for Interactions between Two Major Threats to Colony Health

**DOI:** 10.3390/insects8020048

**Published:** 2017-05-08

**Authors:** Esmaeil Amiri, Micheline K. Strand, Olav Rueppell, David R. Tarpy

**Affiliations:** 1 Department of Biology, University of North Carolina at Greensboro, Greensboro, NC 27402, USA; o_ruppel@uncg.edu; 2 Department of Entomology & Plant Pathology, North Carolina State University, Raleigh, NC 27695, USA; david_tarpy@ncsu.edu; 3 Life Science Division, U.S. Army Research Office, Research Triangle Park, Durham, NC 27709, USA; micheline.k.strand.civ@mail.mil

**Keywords:** honey bee queen, queen quality, honey bee disease, colony health, colony fitness, queen fitness, pollinators

## Abstract

Western honey bees, *Apis mellifera*, live in highly eusocial colonies that are each typically headed by a single queen. The queen is the sole reproductive female in a healthy colony, and because long-term colony survival depends on her ability to produce a large number of offspring, queen health is essential for colony success. Honey bees have recently been experiencing considerable declines in colony health. Among a number of biotic and abiotic factors known to impact colony health, disease and queen failure are repeatedly reported as important factors underlying colony losses. Surprisingly, there are relatively few studies on the relationship and interaction between honey bee diseases and queen quality. It is critical to understand the negative impacts of pests and pathogens on queen health, how queen problems might enable disease, and how both factors influence colony health. Here, we review the current literature on queen reproductive potential and the impacts of honey bee parasites and pathogens on queens. We conclude by highlighting gaps in our knowledge on the combination of disease and queen failure to provide a perspective and prioritize further research to mitigate disease, improve queen quality, and ensure colony health.

## 1. Introduction

Pollination is required for approximately 35% of global food production, and honey bees (*Apis mellifera*) are the only pollinators successfully managed on a large scale [1,2]. Forty percent of invertebrate pollinators are at risk of extinction, and even managed honey bee colonies have been dying at high rates for several decades [3,4,5,6,7]. Continued declines in pollinator health imperil global food security and economic stability.

The death of honey bee colonies is a complex phenomenon that can be attributed to multiple biotic and abiotic factors, as well as interactions among them [6,8,9,10,11]. Pathogens and parasites have been linked to poor colony health and can increase colony losses [12,13,14,15,16]. The ectoparasitic mite, *Varroa destructor*, is considered the main candidate involved in winter colony losses in most managed honey bee populations [15,17,18,19,20,21]. Furthermore, pathogenic viruses are a major threat to honey bee colonies [12], especially those associated with *V. destructor*, including Deformed wing virus (DWV), Israeli acute bee paralysis virus (IAPV), and Acute bee paralysis virus (ABPV) [12,15,19,22,23,24,25,26]. Honey bee mortality may increase when virulent forms of associated viruses are vectored by the varroa mite [27]. The microsporidian *Nosema ceranae* has also been associated with collapse of honey bee colonies in some countries, although its role remains controversial [28,29,30,31]. *Melissococcus plutonius*, the etiological agent of European Foulbrood, is a serious disease of honey bee brood and can lead to the loss of the host colony [32], which has been increasingly observed in Switzerland and the UK [33,34]. *Paenibacillus larvae* causes American Foulbrood, which has historically been the most noxious honey bee larval disease and is also causing economic losses to beekeepers worldwide [35].

In addition to parasites and pathogens, failure or loss of queens has been considered one of the most important factors leading to colony losses [5,15,36,37,38], especially outside of the typical queen-rearing season. “Poor queens” are reported as the primary problem in a number of beekeeping operations, and it consistently ranks among the top reasons for colony failure [38,39]. The queen is the sole reproductive female in a colony, and the presence of a healthy, high-quality queen is essential for colony survival not only because of her ability to lay large numbers of female and male eggs but also because the social coherence of a colony depends on her pheromones [40]. Although queens have a 3–4 year adult lifespan [40], diminished longevity of queens (<1 year) is commonly and increasingly observed [7,39,41,42]. As such, commercial beekeepers typically replace their queens every 1–2 years [43,44,45,46] because of the critical importance of a vigorous queen to colony survival and productivity.

Here, we review the literature on queen quality and the impacts that honey bee parasites and pathogens have on queen health. We aim to highlight the gaps in this area of research to give perspective and priority for further investigations in order to improve queen quality and colony health. Ultimately, we seek to help beekeepers produce and maintain healthy and high-quality queens, in order to promote colony survival and to maintain the economic viability of the apiculture industry.

## 2. Honey Bee Queen Reproductive Potential

The growth, productivity, and survival of a colony, with tens of thousands worker bees, depends in large part on the health and reproductive capacity of its queen and the number of drones with which she has mated [41,46,47,48]. The quantitative and qualitative reproductive potential of a queen represents her “quality”, which results from her genome, her developmental conditions, mating success, and adult environment (including beekeeper management) [49,50,51]. Here, we chose to focus on several fitness-related criteria of queen quality, including physical characteristics and mating success that define reproductive quality in queens and have been empirically linked to colony health and productivity.

### 2.1. Physical Quality

#### 2.1.1. Body Size

Queen weight is a physical characteristic that is critical for evaluating the quality of honey bee queens [52,53,54,55]. Specifically, common measures have been weight at emergence (which is presumably associated with queen’s ovaries; but see [56,57]), weight after mating, and the onset of oviposition [52,58]. Most often, queen weight at emergence is used as an indicator of queen quality, a measure that varies drastically [52,58,59,60]. This variation is influenced by a variety of factors, including the genetic background, the age at which the larva is initially reared as a queen, time of the year, and rearing conditions of the colony [49,52,58,61,62,63,64,65].

The weight of a queen varies at different periods of her adult life [52,54,58]. In virgin queens, it decreases gradually from emergence until mating, with the most rapid loss during the first 36 h. Heavy queens decrease their weight more than moderate and lighter queens [52,58]. After mating, queens start to recover their weights to post-emergence levels [52,58,66]. This seems reasonable because the mating flight(s) requires a lighter body for adequate lift and flight duration, otherwise it can decrease her mating success [67]. After queen mating and the onset of oviposition, a queen’s ovaries start to develop, significantly contributing to an increase in body weight [68]. Queen weight, either at emergence or after becoming reproductive, is significantly correlated with queen attractiveness to worker bees, mating, and reproductive success, although different empirical studies are equivocal on the magnitude of this effect [53,55,60,61,69,70]. Weight is also positively associated with greater acceptance when a new queen is introduced into a foreign colony [70,71,72,73]. Mated and older virgin queens are more attractive to workers and may be more readily accepted than younger virgin queens, because they produce a full queen mandibular pheromone (QMP) profile [74,75], but see [76].

Most reports demonstrate a positive correlation between adult queen weight, the number of mating flights, and overall mating success [52,53,55,77]. While weight at emergence does not seem to be associated with mating [52,69], heavier queens at emergence tend to initiate oviposition later than queens with lower weight after being artificially inseminated [78]. Queen weight also has been shown to correlate with ovary weight, the size and number of ovarioles, the diameter of the spermatheca, and the number of stored spermatozoa [52,53,60,61,77].

Weight is an integrative measure of size and physiological condition. Thus, it could be argued that body weight is the most informative indicator of queen quality. However, external measurements of queen size, such as thorax width, head width, and wing lengths, have also been investigated in correlation with queen reproductive organs in different studies. Results of these studies are equivocal. For example, thorax width was positively correlated with both stored sperm number and mating frequency [53], but no correlation was found between thorax width and ovariole number, ovary weight, or mating number [55,57,77].

#### 2.1.2. Internal Reproductive Organs

In an egg-laying queen, the ovaries occupy the vast majority of the abdominal cavity [40]. The weight of ovaries has been investigated as one of the physical criteria to assess the reproductive potential of honey bee queens [52,79,80]. Ovaries of virgin queens are morphologically different and smaller compared to egg-laying queens [68,81]; because well-developed ovaries are required for egg production, the ovaries of egg-laying queens are about eight times larger than those in virgins [68]. Ovary development occurs soon after mating and is associated with distinct gene-expression patterns in the brain and ovaries, physiological, and behavioral changes in the queen [75,82,83]. The weight of ovaries in a mature egg-laying queen not only depends on the number of ovarioles but also on the number and developmental stage of eggs in them. During winter, egg-laying activity by queens decreases or stops, which results in the queen having smaller and less-developed ovaries [68]. Ovary size and fertility are usually positively correlated [62]. However, under certain circumstances, perhaps due to stress or disease, this relation may not hold [52]. The number of ovarioles can be evaluated at any time during the life of a queen, but it is most reliably scored a few months after mating [84]. In addition to ovariole number, the size of a honey bee queen ovary is determined by the length of the ovarioles, which is more flexible during a queen’s lifetime and reflects her physiological status. Symmetry between the left and right ovaries may [84] or may not [57] be consistent within and among queens or honey bee races [54,57]. Queen ovary size and symmetry are affected by larval nutrition, and during artificial queen production the age of larvae that are transferred into queen cells is critical [60,85], but see [56,62].

After mating, spermatozoa are stored in the spermatheca for the duration of the queen’s remaining life [40]. The number of stored sperm and their viability (i.e., the percentage of live stored sperm) are two critical measures of the reproductive capacity of the queen and her lifespan [59,86]. Therefore, the spermatheca size is another valid measure of physical queen quality, because larger spermatheca can hold more sperm. The spermatheca wall is composed of a single layer of columnar epithelium, lined internally with a thick mucinous cuticle and covered with an extensive tracheal network [84]. Spermatheca size can be measured with or without the tracheal net, and its diameter should be larger than 1.2 mm for high-quality queens [54,84]. The size of the spermatheca in a queen is also influenced by rearing conditions and the genetic source of the queen and is inversely proportional to the age at which the queen is initially reared as larvae [55,62]. Queens raised from newly hatched larvae have larger spermathecae [56,62,80]. This measurement has been used as a direct estimation of the volume and as an indirect estimation of the theoretical maximum number of spermatozoa stored in spermatheca [55,61,77,84]. However, spermathecae are rarely filled completely, as the occupied volume in experimental queens is only an average of 47% [55,77].

### 2.2. Mating Quality

#### 2.2.1. Mating Number

Very early in their lives (1–2 weeks of age), honey bee queens take one or several mating flights [87] to mate with drones. Mating usually occurs at a considerable distance from their natal colonies at so-called drone congregation areas (DCAs) [88,89,90,91], presumably to avoid inbreeding. Honey bee queens are highly polyandrous, with the typical mating number of queens varying between 6 and 26 with an average of 12–14 [53,87,89,92]. Such estimates have typically been obtained using genetic marker analysis (normally microsatellites) applied to the worker offspring of a queen to determine the number of subfamilies (patrilines) and therefore the number of drones with which she mated [53,89,92]. There have been dozens of theories to explain polyandry among insects [93,94]. While there are certainly fitness costs of multiple mating at the individual level (e.g., longer exposure to predation, increased exposure to diseases [95]), several counterbalancing benefits to polyandry exist. Perhaps most importantly, multiple mating increases the genetic diversity within the colony, leading to an increased likelihood that the genetic resources exist within the colony to withstand new biotic or abiotic threats [96]. More specifically, multiple mating has been shown to offer benefits including an increase in the number of stored sperm [53,91], enhanced queen attractiveness [75], enhanced division of labor within the colony [97,98,99], stabilization of brood nest temperature [100,101], improved communication among the workers [102,103,104], reduced incidence of disease, and improved colony fitness [48,105,106,107,108], all of which positively affect colony growth and survival [41,48]. It should be noted that mating number is generally used as a proxy for genetic diversity, if the drones are too inbred or exhibit low genetic diversity, the advantage of multiple mating for the queen and the colony will be muted. Overall, however, increased mating number and the resultant intra-colony genetic diversity of nest-mates is another important criterion for determining the reproductive quality of queens [48,106].

#### 2.2.2. Insemination Success

Queen longevity is inextricably linked to an adequate number of viable stored sperm in the spermatheca, because queens are often replaced once they begin to lay unfertilized drone eggs inside the worker brood nest as a result of sperm depletion [40]. The critical stage of sperm storage occurs immediately after mating [109] when a queen returns to her hive with an average of 10–20 μL of semen (containing 100 million spermatozoa) in the median and lateral oviducts within her genital tract [110]. The vast majority of this semen is discharged, with only 3%–5% of each drone’s sperm actively migrating into the spermatheca where it is stored [110]. The estimated number of stored sperm in a queen’s spermatheca has been used to assess her reproductive quality [53,55]. Woyke [110] considered queens that carried fewer than 3 million sperm to be “inadequately mated”. Different studies have shown that 13.6%–19.0% of commercially produced queens are below this threshold which indicates a serious problem, although significant variation among commercial sources exist [53,55,79,111]. Insufficient sperm supply clearly constitutes a fitness disadvantage for the queen herself as well as her colony, as the total number of sperm decreases with the age of the queen through use and abiotic stressors such as pesticides [112,113].

The viability of spermatozoa is also a crucial parameter of mating and reproductive success, which should be maintained at a high level throughout mating and storage [114]. After sperm storage, sperm cells remain quiescent and the queen provides them with secretions from the spermathecal gland to keep the sperm viable over several years [115,116]. Sperm viability in commercially produced queens showed significant differences across queen producers with an average of 83.7% [55]. Generally, higher sperm mortality is observed in the spermatheca of older queens [112]. Even the movement patterns of the spermatozoa stored in the spermathecae of older queens are slower than those from younger queens [86]. Low sperm viability due to temperature spikes has also been directly linked to colony failure [117].

## 3. Parasites and Queen Quality

Queens are considered to be less susceptible to infections than workers [118]. Queens are continuously attended to and fed by young worker bees [40], which may provide physical and social barriers to protect them from infection [119,120]. In addition to such social immune mechanisms, queens may also be inherently better equipped with individual immune defenses [121]. Nonetheless, queens are susceptible to most of the diseases that infect worker bees, including *Nosema* spp. [28,122,123,124,125], the tracheal mite *Acarapis woodi* [111,126,127,128], and numerous viruses [53,129,130,131,132,133]. Although studies have measured parasite and pathogen levels in queens, only a few studies have investigated the impact of these threats on queen health, performance, and lifespan [119,130,134,135]. Even fewer studies have investigated transmission routes of these pathogens and parasites to the queen [95,136,137,138] and the vertical transmission to offspring throughout the colony [138,139,140]. Due to modern apicultural practices, queens represent an important mode of inter-colonial disease spread even over long geographic distances [141]. Thus, the physiological quality of queens may be affected by diseases but the vector capacity of the queen for diseases is another measure of quality [54,142].

### 3.1. Varroa Mites

The parasitic varroa mite, *V. destructor*, is an obligate ectoparasite of honey bees and generally considered to be the most serious threat for managed honey bee populations [11,16,17,18,143,144]. The impact of varroa mites on honey bee health is particularly severe because varroa can also act as a vector for a number of bee viruses [145,146,147,148]. The act of feeding on bees by varroa directly injects a large number of viral particles into the host [145,147,149], selecting for certain (more virulent) virus strains and causing viral pathologies, such as immunosuppression, weight loss, decreased flight ability, and reduced lifespan [19,150,151,152]. Population increases of varroa mites lead to varroosis in the colony [20], which ultimately leads to colony loss. The reproductive success of a mite is positively correlated with its host’s post-capping developmental duration. Consequently, mites prefer to parasitize drone brood over the workers and queens [153,154] presumably because the drones’ longer development time increases mite fitness. Drone brood suffers from higher varroa parasitism than worker brood because of active choices by the mites based on brood or food odors, or because a slower drone development and more nurse bee visits translate into more opportunities to infest drone cells [155,156].

Contrary to their preference for drones, mites are rarely observed in queen cells. This aversion is likely an adaptive response to the shorter post-capping stage of queen brood (8–8.5 days) and may be mediated by intrinsic differences of larval scents and presence of octanoic acid in royal jelly [154,157]. Because of the short post-capping period of queens, varroa mites cannot successfully complete their reproductive cycle in a queen cell. Therefore, the damage of varroa to queen brood has not been considered important. Similarly, phoretic varroa mites are mainly seen on adult drones and workers but not on the queen, probably due to constant attendance of the queen by workers. Queen infestation by varroa occurs only at extremely high mite prevalence in the colony and the near absence of drone or worker brood [158]. Thus, varroa is generally not a direct concern for queen health or quality but their impact on viruses (see Section 3.4) within a colony poses an indirect threat to queens.

### 3.2. Tracheal Mites

The tracheal mite, *Acarapis woodi*, is an obligate endoparasite of honey bees and was discovered following extensive colony mortality in the UK [159]. This microscopic parasite mostly dwells in the respiratory airways and causes acarapisosis or acariosis disease in adult worker bees [160]. The pathogenic effects of *A. woodi* on individual bees depend on the number of parasites within the tracheae, which can be attributed to mechanical injury, physiological disorders stemming from nutritional loss, damage to tracheal systems, reduction of tracheal airflow and paralysis of flight muscles [161,162]. Colonies with high mite infestation tend to have poor winter survival [163].

Although the concerns about *A. woodi* have decreased recently, studies have demonstrated a wide range of mite prevalence [111,126,127]. Similar to workers, queen infestation is age dependent, with 1-day-old queens carrying on average 6.5 mites and 10-day-old queens only 1.0 mite [128]. The infestation of queens tends to increase with the level of worker infestation around the queen [127,128]. In commercial queen breeding operations, young queens may become infested in mating nuclei if worker bees are already infested, although the results from different surveys vary [53,111]. It is not yet known whether tracheal mite infestation impairs the performance of queens, both in terms of mating or long term productivity and survival. Breeding selection programs have tried to develop resistance to *A. woodi* by improving worker autogrooming, but experimental studies comparing queens from resistant and susceptible lines indicate that the queens from resistant colonies do not have reduced tracheal mite infestation [127].

### 3.3. Nosema Species

*Nosema apis* and *N. ceranae* are two common intestinal parasites, causing Nosemosis in European honey bees [164] by attacking the epithelial cells in the midgut [30,165]. While *N. apis* shares a co-evolutionary history with the European honey bee, *N. ceranae* has more recently been transmitted from its original host, *Apis cerana* to Western honey bees [166]. *Nosema* spp. are obligate parasites and their spores are transmitted horizontally through oral and fecal pathways [167,168]. Recent evidence also suggests that *Nosema* may be transmitted sexually but not vertically [122,168,169,170]. Both *Nosema* species are limited to reproducing in the midgut [165,171], although *N. ceranae* seems to be less tissue-specific and has been found in queen ovaries [122,170]. Symptoms caused by *N. apis* are a large number of dead adult bees in the colony and diarrhea spotting at the hive entrance early in the spring. For *N. ceranae* infections, however, no conclusive evidence of strong seasonal patterns exists [28,30]. Its symptoms are increased foraging duration, decreased flight frequencies, decreased immune functions, and general stress in worker bees that reduces longevity, leading to colony depopulation and collapse [30,172,173,174].

Queens, like other members of a colony, can be infected by both *N. apis* and *N. ceranae* [122,124], and *N. ceranae* has even been detected in larval queens [122]. Most transmission of *Nosema* spp., presumably occurs during the adult stage, including mating, although antimicrobial molecules in drones’ semen are able to kill *N. apis* spores and reduce the risk of disease transmission during mating [169]. Nosemosis in queens causes aberrant physiology, as well as similar gut lesions and metabolic costs as in workers [135,167]. In addition, queens infected by *N. apis* start oviposition later than healthy ones [175,176], display changed pheromone production [135] and in extreme cases their oocytes degenerate leading to infertility [134]. *N. apis* infection may severely reduce queen lifespan to an average of nearly 50 days, resulting in queen supersedure [176], but not in all cases [127]. Compensatory increases in the level of vitellogenin and other antioxidant enzymes occur in infected queens [135]. These counterintuitive changes may be protective mechanisms that are too costly in the long-term for the infected queen to survive. Recent surveys indicate that infection levels in commercialized produced queens are far less than during past decades [53,55,111].

### 3.4. Viruses

Viruses in honey bees, like in all organisms, are non-living, opportunistic, and obligate intracellular pathogens that require the host cellular machinery for transcription, translation, and replication [177]. Viral infection may cause direct effects on the morphology, physiology, and behavior of honey bees [24,25,178]. Thus far, 23 positive-strand RNA viruses have been reported to infect honey bees [179,180,181]. The most commonly studied honey bee viruses can be grouped into the families *Dicistroviridae* (Black queen cell virus, BQCV; ABPV; IAPV; and Kashmir bee virus, KBV), *Iflaviridae* (DWV; Slow bee paralysis virus, SBPV; and Sacbrood virus, SBV), and an uncategorized family (Chronic bee paralysis virus, CBPV) [179].

Deformed wing virus (DWV) is a nearly omnipresent and persistent virus, associated with varroa mite infestation [24]. However, prior to or in the absence of varroa, DWV can also be transmitted through other pathways between and within castes [137,140,146]. In the absence of varroa, sexual and vertical transmission of DWV may have been more important. In the post-varroa era of apiculture, drones from colonies with high mite infestations show high levels of DWV infection [182]. Infected drones are able to fly and frequently reach drone congregation areas to mate with young queens [95,136]. DWV has been detected in drone samples from DCAs, the queen “mating sign” (the remaining endophallus of her last mating partner), and semen collected from queen spermathecae, all of which indicate venereal transmission of DWV [95,136,137]. However, it is yet unclear if this is the main mechanism by which uninfected queens become newly infected. It has been also found that the virus is able to transmit vertically from an infected queen to her offspring [138,139,140,183]. Heavily infected workers usually emerge with the symptomatic crippled-wing syndrome and shortened abdomens, resulting in premature mortality [24,184]. The virus has been detected in older queens as well as young queens. The latter have usually a lower virus prevalence [53,129,133], suggesting that the virus replicates in adult queens. DWV has been found to infect the head, fat body, gut, and ovaries of queens [95,130,182,183], although crippled wings have seldom been reported in queens as a consequence of DWV infections [185]. A high virus titer in reproductive tissues can lead to ovarian degeneration or possibly affect stored sperm viability [130]. Therefore, a DWV-induced decline of reproduction quality may seriously affect colony performance, productivity, and queen supersedure.

Chronic bee paralysis virus (CBPV) was the first isolated and described virus causing chronic bee paralysis in adult worker bees [186]. CBPV can be transmitted through the fecal–oral pathway or via contact between adult bees when healthy bees are crowded together with infected individuals [187]. An outbreak of CBPV can lead to severe mortality of the workers and eventually to a collapse of the colony even within a single active season [188]. Often, the queen and a few workers are the only remaining individuals in collapsed colonies [189]. Several studies have detected a low prevalence of CBPV in surveyed queens [131,133,183]. Experimentally infected queens show the same symptoms as worker bees with trembling of legs, spread and disjunct wings, and a bloated abdomen full of hemolymph with a dilated honey sac. Queens are as susceptible to CBPV as workers [119].

Acute bee paralysis virus (ABPV) and Israeli Acute Paralysis Virus (IAPV) are closely related viruses, commonly existing as covert low-titer infections [25]. In association with varroa mites, they are additional factors that promote colony collapse [23,132,147]. ABPV and IAPV are virulent pathogens that cause paralysis, trembling, and rapid death of workers in 1–2 days after infection [25]. These viruses have been found in different developmental stages and castes [132], although high titers have been rarely detected in queen surveys [129,131,133,183]. As such, the impact of ABPV and IAPV on queen health has not yet been well studied. The only investigation by in situ hybridization of IAPV showed specific detection of the IAPV in the egg, gut, ovaries, and spermatheca of infected queens [132].

Sacbrood virus (SBV) causes brood disease of the honey bee, but it has also been reported from adult honey bees without any obvious sign of disease [190]. The virus mostly transmits through the oral-oral pathway from adult worker bees to the larvae, and clear symptoms appear a few days after capping [178]. Infected larvae fail to pupate, and ecdysial fluid accumulates beneath their unshed cuticle, the larvae change color from pearly white to pale yellow, and shortly after the larvae die [190]. The virus also has been detected in queens, mostly in the ovary and eviscerated body (i.e., non-specific tissues) but how it affects queen health or whether it can be vertically transmitted is not well studied [131,139,183].

Black queen cell virus (BQCV) was first isolated from dead queen pre-pupae and pupae sealed in their cells that had turned dark brown to black along the walls of the cell [191]. It is consistently one of the most common viruses detected using laboratory techniques in adult honey bees worldwide [177]. BQCV infection is often observed in queen-rearing colonies, attacking queen larvae and pupae. Coinfection of workers with BQCV and *N. apis* within a colony results in increased worker bee mortality [192], although data to support synergistic interaction between BQCV and *N. ceranae* are controversial [193,194]. The virus has been detected with high titers in collapsed colonies [13], and it has been detected in honey bee queens mostly in gut, feces, and ovaries [131,183].

In sum, it is clear that several viruses cause overt problems for queen health but more commonly viruses remain asymptomatic and can be efficiently transmitted by queens into the next generation [133,139]. Thus, viruses should be under selection for decreased virulence towards queens, but little data are available to test this prediction. Practically, it is unknown how the presence of covert viruses in queens affects them because non-invasive methods for virus detection in honey bees remain to be developed. Many other viruses are also known to exist in honey bee colonies, however they remain relatively poorly characterized and little or nothing is known about their effects on queens. This lack of even simple descriptive information makes them important targets for further empirical investigations, especially with regards to their connection to queen and colony health.

## 4. Conclusions

We have explained above how to quantify queen quality and how different diseases can affect queen reproductive potential. In turn, high quality queens with high reproductive potential produce colonies that exhibit high growth and survival [46]. Young and healthy queens produce a solid brood pattern, facilitating brood care that can prevent disease, and a greater proportion of this brood will develop into healthy workers to replenish dying workers [44,195]. These colonies are able to store more honey and pollen throughout the year in comparison to the colonies headed by low quality queens [46], which translates into better winter survival. Moreover, reproductive quality influences queen mandibular gland pheromone profile and influences colony cohesion [196]. Replacing the queen in *Nosema*- and CBPV-infected colonies with a young, healthy, and productive queen has been recommended to maintain the colony homeostasis [188,197] and presumably is also advisable when other diseases are detected. Through direct and indirect effects a healthy, high-quality queen can mitigate disease effects on colony fitness or even make colonies more resistant to disease [105]. However, more research to demonstrate these interaction effects is clearly needed.

Our review focuses on the intersection between queen reproductive potential, and honey bee diseases. How these interactions manifest themselves will be significant for understanding colony health problems and the means to mitigate them. Based on our review, we believe the following questions should be prioritized for future investigation to help elucidate these interactions and improve colony health. (1) How do queens initially become infected? There are multiple routes that have been identified (orally during juvenile and adult life stages, as well as sexually), but we lack in many cases a comprehensive understanding of exposure, within-queen pathways, and key stages of infection in queens. If we can identify the means by which queens become infected, then we may be able to identify the means to avoid infection of queens and their colonies. (2) What triggers viral replication so that a previously chronic low level (asymptomatic) infection becomes harmful? Several studies have shown that viruses can be detected as a low, covert level in honey bee queens [129,133], but the effect of different stressors on the queen to trigger the viruses to become overt and harm the queen is not clear yet. (3) What is the collective decision-making process of workers to replace sick queens through supersedure? Researchers have developed a comprehensive model for the internal and external conditions that trigger swarming behavior in colonies [198], but we lack a similar theoretical context for non-swarming queen replacement. Developing such a model is a top priority for research, because early or failed supersedure attempts are an increasingly common problem experienced by beekeepers, and such events lead to loss of colony productivity and increased mortality. (4) What are the specific, various phenotypes of “poor queens”? There are many “queen problems” experienced by beekeepers, but the mechanistic basis is unclear. It will be critical to tease apart the various phenotypes of “poor queens” and explain their underlying causes, as it is possible if not likely that different symptoms of queen problems can be attributed to different factors. (5) What is the effect of a queen’s microbiome on her health and disease resistance? The typical microbiomes of workers and hive substrates have been described (e.g., [199]; reviewed in [200]), but our understanding of queen microbiota is relatively lacking [201,202]. One particularly interesting question is the effect of the queen on the colony microbiota and vice versa (the effect of diverse colony parameters on the queen’s microbiota). In the light of the increased interest in probiotics, this area will also have direct implications for hive management. (6) How does disease mechanistically affect the queen’s pheromones, chemical signaling, and reproductive physiology? Investigating the interactions between queen physiology and disease is critical for understanding which resistance factors are more important than others. Functionally, these interactions are likely going to be expressed through the pheromonal signaling of the queen and their perception by the workers. Determining how infection may influence a queen’s signals, the workers’ perception of them, or both will demonstrate how colony cohesion is ultimately affected by disease. Finally, (7) How does a potential shift to microbreeding affect queen quality and spread of disease? Currently, the majority of queens in the US are produced by relatively few commercial queen producers [203], which raises concerns about a lack of genetic diversity and the spread of certain diseases [141,203], particularly those that are transmitted vertically. There are currently many efforts to promote smaller-scale, localized production of queens (“microbreeding”) to address supply and favor locally adapted genotypes (see [204]). However, there is little if any information about whether such efforts may help bolster genetic diversity, reduce disease prevalence or intensity, or otherwise have a positive effect on overall queen quality. Potentially, new metrics of queen quality that include disease resistance or immune measures are needed to guide breeding efforts.

In sum, the interactions between queen health and honey bee diseases have not been sufficiently studied because their importance was underestimated. However, many unanswered questions in this area provide fertile grounds for fundamental biological research and the possibility to apply insights to improve honey bee health.
